# Why functions are not special dispositions: an improved classification of realizables for top-level ontologies

**DOI:** 10.1186/2041-1480-5-27

**Published:** 2014-06-02

**Authors:** Johannes Röhl, Ludger Jansen

**Affiliations:** 1 Institut für Philosophie, Universität Rostock, 18055 Rostock, Germany; 2 Philosophisches Seminar, Universität Münster, 48143 Münster, Germany

**Keywords:** Function, Disposition, Role, Process, Realizable, Artefacts, Top-level ontology, BFO

## Abstract

**Background:**

The concept of function is central to both biology and technology, but neither in philosophy nor in formal ontology is there a generally accepted theory of functions. In particular, there is no consensus how to include functions into a top-level ontology or whether to include them at all.

**Methods:**

We first review current conceptions of functions in philosophy and formal ontology and evaluate them against a set of criteria. These evaluation criteria are derived from a synopsis of theoretical and practical requirements that have been suggested for formal accounts of functions. In a second step, we elucidate in particular the relation between functions and dispositions.

**Results:**

We argue that functions should not be taken as a subtype of dispositions. The strongest reason for this is that any view that identifies functions with certain dispositions cannot account for malfunctioning, which is having a function but lacking the matching disposition. As a result, we suggest a cross-classification of realizables with dispositions supervening on the physical structure of their bearer, whereas both functions and roles also have some external grounding. While bearers can survive the gain, loss and change of roles, functions are rigid properties that are essentially connected to their particular bearers. Therefore, *Function* should not be regarded as a subtype of *Disposition*; rather, the classes of functions and dispositions are disjoint siblings of *Realizable*.

## Background

The ascription of functions is central to biology as well as to psychology, technology and engineering. However, realizable entities like functions, dispositions and roles are notoriously difficult to understand and there is no consensus how to model them within a top-level ontology. The more general debates in the philosophy of biology and technology also offer several theories of function with their respective advantages and shortcomings. Because of the diversity, plurality and ambiguity of function concepts in, e.g., engineering, some authors have claimed that there are many different function concepts which are only connected by “family resemblances” [1]. Even if this is true, it will nevertheless be useful for the representation of scientific statements about functions to focus on some of the more important uses of the word and fix these within a formal ontological framework. One particular challenge is whether there can be an overarching meaning of the term ‘function’ both for biological and artefactual functions.

Another challenge we will address concerns the relationships between functions and other kinds of entities like dispositions and roles in formal ontology. That these relations are not at all clear is witnessed by BFO, the Basic Formal Ontology [2,3]: BFO versions up to 1.1.1 contain the categories *Disposition*, *Function* and *Role* as jointly exhaustive and pairwise disjoint children of the category *Realizable*, but in the transition to the new version BFO 2 it is planned to position *Function* as a subtype of *Disposition* (cf. Table 1 for more details).

**Table 1:** Definitions of the children of realizables in BFO 1.1.1 and 2 (‘Graz release’)

**Definition in BFO 1.1.1**[2]	**Definition in BFO 2**[9]
**Disposition** = A realizable entity that essentially causes a specific process or transformation in the object in which it inheres, under specific circumstances and in conjunction with the laws of nature. A general formula for dispositions is: X (object) has the disposition D to (transform, initiate a process) R under conditions C.	b is a **disposition** means: b is a realizable entity & b’s bearer is some material entity & b is such that if it ceases to exist, then its bearer is physically changed, & b’s realization occurs when and because this bearer is in some special physical circumstances, & this realization occurs in virtue of the bearer’s physical make-up.
**Function** = A realizable entity the manifestation of which is an essentially end-directed activity of a continuant entity in virtue of that continuant entity being a specific kind of entity in the kind or kinds of contexts that it is made for.	A **function** is a disposition that exists in virtue of the bearer’s physical make-up and this physical make-up is something the bearer possesses because it came into being, either through evolution (in the case of natural biological entities) or through intentional design (in the case of artefacts), in order to realize processes of a certain sort.
**Role** = A realizable entity the manifestation of which brings about some result or end that is not essential to a continuant in virtue of the kind of thing that it is but that can be served or participated in by that kind of continuant in some kinds of natural, social or institutional contexts.	b is a **role** means: b is a realizable entity & b exists because there is some single bearer that is in some special physical, social, or institutional set of circumstances in which this bearer does not have to be & b is not such that, if it ceases to exist, then the physical make-up of the bearer is thereby changed.

In this paper, we will try to meet these challenges. For this purpose, we will review different philosophical theories of functions and evaluate them with respect to a set of desiderata for function theories. In the remainder of this section, we will give some background information on the current state-of-the-art representation of functions, reviewing work on this topic published by the research groups that developed the top-level ontologies BFO, DOLCE and GFO [2,4,5].

### Functions as realizables in BFO

In the older versions of BFO, *Function*, *Disposition* and *Role* are sibling subclasses of the class *Realizable dependent continuant*[6]. This common superclass implies that instances of any of these three classes share the following characteristics:

– They are continuants, i.e. they are wholly present at every time of their existence.

– Like qualities, they are (specifically) ontologically dependent on an independent continuant (some material thing or system) that is their bearer.

– They are realizable, i.e. they are by definition connected to certain types of processes such that instances of such a process type can be realizations of the realizable entity in question.

– When they are realized, their bearers are participants of their realization processes, i.e. of the processes they are roles, dispositions or functions for.

Note that realizables do not need to be (always or ever) realized [7], as, e.g. in the case of a safety mechanism, the function of which will only be realized if certain conditions obtain (and they may never obtain). Also note that several other types of realizables are conceivable like, e.g., propensities, tendencies, abilities, capacities, virtues and vices [8]. Arp and Smith [6] conceptualise the specific differences between functions, roles and dispositions as follows (cf. Table 1): The realizations of functions and dispositions take place “in virtue of the bearer’s physical makeup”, whereas a role is “optional”: It does not reflect the intrinsic structure of their bearer but a “natural, social or institutional set of circumstances”. Functions are distinguished from dispositions by the additional condition that the function bearer possesses the physical structure that grounds the function because of how it came to be there in the first place: In the case of artefactual functions by intentional design and production or in the case of biological functions by a history of evolutionary selection. In BFO 2 the relation of functions and dispositions was revised: functions are now a subclass of dispositions [9], as detailed in Table 1. Although we sympathize with BFO as a top-level ontology and will later on use its other categories, especially the fundamental disjoint classes of continuants and occurrents and their relations, we find that its treatment of functions is in need of some improvement and clarification and we will later suggest a way to do so.

### Functions and flows in DOLCE

While the top-level ontology DOLCE [4] neither includes functions nor dispositions or roles in its core version, there are several suggestions for a formalisation of engineering functions within the DOLCE framework [10-12]. However, their formalisation starts from a very specific technical approach that focuses on “flows” of materials, energy or signals. Functions are then, basically, what relates certain input and output flows. While being useful in engineering, this approach is mostly orthogonal to the debates on the functions of biological entities. One main goal of these authors seems to be the integration of the “flows” in the DOLCE ontology and they accordingly classify different sorts of flow as various types of process-like entities in DOLCE (states, processes etc.) and characterise their dependencies and relationships with the continuants involved (which are called “endurants” in DOLCE). The relationships between functions, dispositions and roles that concern us in the present paper are not discussed. We will not distinguish between different types of processes as inputs or outputs for functions for two reasons: First, DOLCE’s subtypes of processes are heavily description-relative and, hence, linguistic artefacts. Second, this model is not quite as natural for biology as for applications in engineering. We can state that the function of the eye is to see without being able to specify input and output flows. The point is not that one could not conceptualise “seeing” in terms of “flows” of visual and neurological signals, but that it is not necessary to understand the function of biological organs in these terms. Consequently, analyses of functions in the philosophy of biology do not normally refer to input and output flows (though it can be argued that such ideas are highly relevant to certain strands of psychology or functional theories of the mind). Let us go through an example: The function of an animal’s legs is locomotion, so the actual locomotive process of the animal is the realization of the function and this motion could also be called an “output”. But what is the input? Is it some neurological signal from inside the animal or some stimulus from the environment? Or is it a flow or change of energy in some cell or neural pathway? In order to understand that the function of legs is locomotion, the question about the input seems to be irrelevant. In any case, such a fine-grained approach with respect to input or output flows does not add anything to the main question of our paper. We take up this point again in the Methods section.

### Work from the GFO-Group

An elaborate and somewhat complicated model of functions has been developed by researchers from the OntoMed group that has produced the General Formal Ontology (GFO) [13,14]. According to GFO, like in BFO, functions have realizations which are usually processes, but unlike in BFO, these realizations could also be continuant entities. Burek et al. characterise a function by three “function determinants”, in particular:

(1) the preconditions for the realization of this function,

(2) the goal affected or brought about by the function (or its realization, respectively),

(3) the “functional item” which corresponds to the role of the material bearer of the function as the participant in the realization of the function.

In addition to functions and realizations, they introduce “realizers”. A realizer is the actual particular entity that plays the role of the “functional item”. Furthermore, they distinguish the realization of a function (usually a process) from the “goal” which is a “state of the world” that is reached by means of the realization starting from the precondition requirements. The authors illustrate these components using the example of oxygen transport in the human body. The realization of this function is an actual process of oxygen transportation and it has as precondition the presence of some oxygen at location A and as goal state the presence of some oxygen at location B. The “functional item” is denoted by the nominalised role term “oxygen transporter” and in the case of oxygen transports in the human body this role is played by instances of red blood cells, which are, thereby, “realizers” of this function. Thus, according to this approach, roles are, to some extent, always involved when we deal with functions and roles are explicitly dependent on “role contexts”. However, note that these “roles” are different from BFO roles and should not be confused with them.

Burek et al. also distinguish “dispositional function” from “actual function” where the latter applies only to functions that are actually realized. This seems to be redundant as it ignores that this modal feature is captured already by the distinction between functions as realizables and their realization processes. Despite its elaborate apparatus, this approach does not help much to distinguish functions from roles and dispositions because all three terms are used to characterise functions. Elsewhere Burek seems to conceive of functions as a subclass of dispositions, stating that only the dispositions for “effects of an item which are related to some (pre-) defined system of functions and goals” are its functions [14]. This statement could be taken to mean that functions should be understood as a special kind of disposition. In any case, it seems that intentions of agents (like designers and users of an artefact) choose the functions of an item and functions are characterised as intentional entities ([14] definition 67, p.157) and, therefore, agent-dependent (or community-dependent) and subjective. Altogether, this account seems to be better suited to technical functions. Still, many of its features are perfectly compatible with the BFO account, which we try to improve upon. The central structure of functions as having bearers and being realized in processes with the bearers as participants is very similar. BFO refrains from some subtleties like the explicit consideration of the preconditions for the realization of a function or the distinction between a goal-state and the realization as a process leading to a goal state. A possible reason for abstracting from these distinctions is the actual talk about functions in much of biology (and the philosophy of biology) where the process of blood-pumping as a realization of the heart’s function is normally not contrasted with a distinct goal state like, e.g., the distribution of nutrients and oxygen in the body achieved by the blood-pumping. For many organic functions, the preconditions for realization are often implicitly presupposed. For example, the function of the heart is to pump blood and in order to realize this function it has to be part of a living body, be connected to arteries and veins, and enough blood to be pumped has to be present. All of these are, of course, features of a physiological organism. Biologists do describe these from the point of view of the whole organism and not for each organ separately. To spell these conditions out would be a tedious and possibly endless endeavour: In order to play a part in locomotion from A to B, legs must be part of an organism situated at A, but not at B, there must not be a hindrance between A and B, it must be possible to be located at B, etc. All this is true, but of no specific interest to the biologist. To conclude, the approach by Burek et al. seems to be compatible with the BFO approach in its main features, but does not help much with the problems of distinguishing and relating functions, roles and dispositions.

## Methods

We will proceed in two steps in this paper. First, we survey philosophical theories of functions and evaluate them against a set of requirements. The theories evaluated are mostly theories of biological functions, but we also look at some theories for artefact functions. In the discussion section we take up the results of this survey and take a closer look at the relation between functions and dispositions.

The requirements used as evaluation criteria will be detailed in this section. In the literature, several lists of requirements or criteria for a theory of functions have been suggested. As will become clear, we will not use all these criteria, but need to pick out a coherent subset of criteria that we will use in this paper.

We extract from Artiga [15] for biological and from Houkes and Vermaas [16] for artefactual functions the following list of adequacy criteria for function theories (somewhat adapting them to our own terminology):

(1) **Teleology**: The function should have a central role in the explanation of the existence of the function bearer [15].

(2) **Restriction**: Proper or essential functions of a thing can be distinguished from its accidental functions or transient effects [15,16].

(3) a. **Normativity**: The performance of a function can be evaluated as better or worse according to a norm given (at least implicitly) by the function ascription [15].

b. **Malfunctioning**: A thing can have a function, although it fails to perform according to this function occasionally or even permanently. Failure to properly perform according to one’s function can be a case of malfunctioning or of non-functioning [16].

(4) a. **Avoidance of epiphenomenalism**: Functions should be determined by current performance of its bearer, not mainly by causally inert historical facts like its (evolutionary or cultural) history or a mere ascription by its producers, users, or observers [15].

b. **Support**: The physical structure of the function bearer supports its function, even in the cases of accidental functions or dysfunction [16].

(5) **Innovation**: Novel functions can be ascribed correctly to innovative artefacts [16], but also to newly evolved organisms.

There are some tensions between these desiderata [17] which are also acknowledged by the authors who proposed them. Artiga argues that the normativity criterion and avoidance of epiphenomenalism cannot be satisfied at the same time because (3) implies that function ascriptions should somehow be independent from the actual properties or activities of the function bearer while (4a) explicitly denies this [15]. Likewise, Houkes and Vermaas state a tension between the malfunction criterion (3b) and the support criterion (4b) for their theory of artefact functions [16]. They claim to have solved this for artefactual functions. This solution is based on, firstly, an optimistic view of the rationality of designers, users and other ascribers of functions who would not, or so they assume, assign unsupported functions and, secondly, on a history of maintenance and repair that makes a distinction between non-function and malfunction easier. But it might well be that not all of the criteria can be satisfied simultaneously and that some may have to be relaxed in any theory of functions.

Normativity is one of the most crucial, but also most contested desiderata for functions. To assert that something has a function or that a function can be performed better or worse, we need some kind of normative dimension. Franssen points out that, as a biological concept, it would have to be naturalist and, thus, cannot be normative in the full-blown moral sense [18], but for the normative dimension of functions it should suffice that it accounts for the fulfilling (or missing) of purpose in a naturalist way [19]. Franssen suggests that we could understand the normativity of functions in a deflationary way; that is, in terms of the rational expectations we have with respect to the functions of organs or artefacts. However, this kind of normativity ascription is too weak because it lays no special claim to functions in particular. We form these rational expectations also with respect to a dropped stone that “is supposed to” fall (thereby obeying a law of nature). Furthermore, Franssen argues that, in biology, functions are attributed more widely than the associated normative evaluations [18]. Many traits or behaviours of living beings can be described as having functions in the evolutionary sense, but are usually not evaluated in the way organs (as quasi-tools of an organism) are. However, we can ignore these borderline cases and still acknowledge that we need the normativity in the case of organs or more generally for all subsystems of organisms to which functions are ascribed and which are evaluated with respect to their performance, as it is clearly the case in medical contexts. These use-cases show that we do need to deal with the normative aspect and, therefore, we will retain this criterion at some expense of criterion (4).

We supplement these criteria with some requirements for an ontology of functions proposed by Burek [14]:

(6) **Continuants**: Functions should not be conflated with their realizations. While functions (and other realizables like dispositions and roles) are continuants, i.e. existing wholly at any point of time during their existence, their realizations are processes that stretch out in time.

(7) **Bridging of Domains**: An ontology of functions should account for both artefacts (devices) and non-artefacts.

We regard it as an asset of a theory of function if it can account for biological and engineered function as uniformly as is permissible given the differences between the two domains. While (6) is fulfilled by all approaches in our survey, it is acted against by such a prominent system as the Gene Ontology [20], which does not clearly separate functions and activities because the term *Molecular function* is used both in the sense of an activity and in the sense of a capability: “Molecular function is defined as the biochemical activity (including specific binding to ligands or structures) of a gene product. This definition also applies to the capability that a gene product (or gene product complex) carries as a potential”. [21] That functions themselves can sometimes be processes has also been suggested by Kitamura and Mizoguchi [22], who talk about processes as “actual functions”. However, what they call an “actual function” corresponds rather to functioning than to function, i.e. to what we (with BFO) call a realization of a function. Only their “capacity function” corresponds to what we call “function” proper. Thus, while they use the term “function” for processes, they do not claim processes are functions in the same sense as the functions ascribed to devices.

Hence, we strongly support (6) and (7), but we will not consider the following desiderata (8) and (9), which have also been suggested by Burek [14]:

(8) **Process functions:** Processes can be bearers of functions.

(9) **Decomposition**: An ontology of functions should support functional decomposition, i.e. the analysis in terms of sub-functions.

Criterion (8) is contentious, as it is in direct contradiction to our preferred framework BFO where only independent continuants can be bearers of functions. We do acknowledge that natural language does, in fact, attribute functions to processes like in the following examples:

A. “His knocking at the door had the function to cause someone to open the door.”

B. “The pumping has the function to keep the blood circulating.”

However, surface grammar might be deceiving. Example (A) is a description of an intentional action and the agent knocking at the door performs this action because of a certain purpose. This shows that it is not necessary to ascribe functions to processes because this comes down to the ascription of intentions or plans of persons participating in these processes. In example (B), the function to keep the blood circulating can, as well, be ascribed to the heart, which is an independent continuant, as BFO requires. The heart can also be said to have the function to pump blood and given the heart’s canonical location within a circulatory system, it fulfils the former function by realizing the latter: It keeps the blood circulating by pumping it. Hence, in these cases, we have an instrumental or causal relation between two processes. While this is an important feature to be modelled, we hesitate to deal with it together with the functions of independent continuants.

We do not consider (9), as decomposition is orthogonal to the relationship between functions and dispositions and, thus, not relevant to the topic of this paper. Decomposition seems to be of interest mainly in the technical domain and is hardly discussed for bio-functions in applied ontology, but it can, of course, be extended to this domain. This is nicely shown by the heart example that we just discussed: The heart supplies oxygene to the body by circulating the blood, and it keeps the blood circulating by pumping it (cf. the hierarchies of biological functions in [23]). Of course, composition of functions cannot be analogous to the mereological composition of the whole by its parts because the function of the whole is not simply the sum of the functions of its parts. Nevertheless, a function of a whole often depends on the functions of its parts, but the composition relation for functions is not straightforward and would be a different topic. While functional decomposition is compatible with our approach, it is not relevant for our present purposes. For these reasons, we will use the criteria (1)–(7) only.

## Results

### Survey of philosophical theories of functions

The concept of a function has provoked a lively debate in the philosophy of biology, of mind and of engineering. Several accounts have been proposed to illuminate the nature of biological functions; see [24] and [25] for recent contributions to this debate. We will now survey the most important suggestions and evaluate them with respect to the desiderata given above.

Among the many different conceptions and theories of functions, three main classes of theories may be distinguished. Like Franssen [18] and Artiga [15], we start by looking at (a) causal contribution theories of functions and (b) etiological accounts. We add to this by looking at (c) intentional accounts and two further developments of the intentional account, namely (d) the ICE theory that combines intentional, causal and etiological elements, and the (e) fictionalist account that extends the intentional account to biological functions.

### Causal contribution theories of functions

Causal contribution theories take criterion (4) as sufficient for the ascription of a function. They are also called “dispositional” [15], “causal role” [26], “goal-contribution” [27], and “systemic” [28] accounts of functions. They all have in common that the function of a thing is linked to the present causal contribution of the function bearer in a certain context. The most straightforward is the simple causal role analysis. According to this analysis, “X has function F” simply means that X does causally contribute to some output O of a complex system S [26,27]. A well-known problem of this account is that it is extremely broad and admits many unintuitive functions: It implies, e.g. that clouds could be ascribed the function to produce rain because they undoubtedly have a central causal role in the production of rain (more examples and further criticism in [27]). Thus, the simple causal role account fails the teleology criterion and no distinction between essential and accidental functions is possible. To ameliorate this, further conditions have to be added in order to narrow down possible functions of a thing. As functions are intuitively connected either with some intention, as in artefactual functions, or a (not necessarily intended or conscious) goal in biological functions, Boorse’s “general goal-contribution” approach [27] could be considered the “minimal core” of the concept of a function with system S, system part X and goal G:

X performs function Z in the G-ing of S at t if and only if at t, the Z-ing of X is a causal contribution to G.

But this still does not satisfy all of the criteria. First, Boorse’s definition does not capture functions as such, but only their realizations. Moreover, Boorse-functions could be performed only once or accidentally and fulfil this definition which would usually not be what we mean when we ascribe a function. (A formal-ontological characterization of such a goal-contribution approach has been sketched in [29]). Boorse still has to rely on distinctions like the “normal” function of a type as opposed to accidental functions or deviations of single tokens to avoid those counterintuitive accidental functions. We will come back to the type/token relation as a source of normativity below. An even more challenging problem for this account is posed by malfunctioning because if X does not perform Z (i.e. if function Z is not actually realized) and no actual causal contribution takes place, we cannot ascribe a function at all. Therefore it is not clear how malfunctioning should be handled within this framework.

The concept of a systemic function, as suggested by Mizoguchi et al. [28], is very similar. They introduce a “systemic context” that structures a system into a nested hierarchy of subsystems and components and assigns a specific behaviour to the system and its components in order to define an object’s “systemic function”:

An object A performs a systemic function within a systemic context C, if and only if there is a system S such that:

(1) C is a systemic context for S,

(2) according to C, A is a component of a subsystem of S,

(3) the goal of this subsystem is to realize the goal of C, and

(4) some behaviours of A play the (functional) role determined by C.

This definition can be applied both to biological and to technical functions. To give a biological example, a systemic context C for the human liver (=A) would be the human digestive system (goal: digestion of food and extraction of nutrients) and within the subsystem of fat digestion the function of the liver is the production of bile. The point is that the systemic function is context-dependent in a specific way, i.e. via a system its bearer is part of. Hence, the function of a thing can change depending on the system it is a component of. In an earlier paper [22], Kitamura and Mizoguchi discuss the example of a heat-exchanging device. This device shows as characteristic behaviour a process of heat transfer that leads to a temperature change in some fluid. This behaviour can serve different functions: It can either have the function of heating by “giving heat” or the function of cooling by “removing heat”. Kitamura and Mizoguchi, therefore, call the role of the behaviour which the device plays in the respective teleological context (here: heat exchange) the “actual function” of the device. Depending on context, this will be heating or cooling. Their expression “actual function” corresponds to what we call “realization of a function” and their “capacity function” corresponds to our “function”. If we follow the idea that the realization gets its role depending on context, we could say that the device has the capacity (=disposition) for the realization heat-exchanging, but can have the (capacity) function of heating or cooling depending on the context. Thus, the context-dependence of the realization of a function can also be claimed for the function as a realizable entity.

In this way, the proper function is fixed by the respective context and can be distinguished from accidental functions. Therefore, the accidental/essential distinction can be maintained by making functions context-dependent: “all systemic functions are essential *with respect to the systemic context*” [28] (italics in the original). However, it is not clear how the systemic context can be fixed without recourse to other factors. Intentional design is such a factor which Kitamura and Mizoguchi [22] take as a standard example for such a context.

To summarize: The causal contribution approaches can account well for the support criterion because functions are closely tied to the dispositions of their bearers and their realizations. They have no problem with novel functions because nothing is said about the history of the function bearer; they avoid epiphenomenalism because the actual performance is central. On the other hand, the problem of accidental and essential functions can only be dealt with by embedding the bearer in a system, which, in turn, may lead to the problem of determining the proper context of a function and, thus, to circularity, making the proper context depend on the proper function and vice versa. A central problem for this account is the possibility of malfunctioning, for if function is taken to be identical with the actual causal contribution of the function bearer, it is not clear how something can both have a function and not perform adequately and it seems very hard to distinguish malfunction from the absence of function. We will come back to this later, but we already note here that many authors do not employ the idea of realizables and, therefore, do not distinguish as sharply between the having of a function (or a disposition) and the actual taking place of the respective realization process. In the approach that takes functions as realizable entities, it is clear from the outset that something may have a function without actually or ever performing it. This does justice to the fact that a function ascription is not tied to actual performance and, thus, rejects the causal role criterion as sufficient for a function ascription, but this alone is not sufficient to explain malfunction.

### Etiological theories of functions

As an alternative to causal contribution theories, etiological accounts of biological functions have been suggested [30,31]. While the causal theories of functions which we discussed so far focus on the effects of functions, etiological theories focus on their causes: The core idea of etiological theories of functions is that a function of X plays a relevant role in an explanation of why X exists in the first place. In the present section we will discuss only those etiological accounts that refer to non-intentional causes, while we will discuss intentional accounts of functions in the next section. In non-intentional etiological accounts, a function is taken to be dependent on the history of the biological kind whose instances are bearers of the function in question (or, as some prefer, on the history of matching “reproductively established families” [30]), i.e. on the series of evolutionary precursors of a present function bearer. It is evolutionary selection that causally explains the existence of the functional parts in the first place. Thus we can phrase the etiological account of biological functions as follows:

Instances of a biological kind X have the function to do F if and only if the F-ing of the instances of X has in the past been causally responsible for the positive selection of X and, thus, indirectly for the present existence of instances of X.

Such an etiological approach can deal with the most salient difficulties of the causal contribution accounts. Essential functions can be distinguished from accidental ones by referring to the selection history of the trait; furthermore, this history is determined by salient effects of earlier versions of the trait, so no external goal or context seems necessary. Since performance of a function is clearly distinguished from having a function, something can have a function (due to its history), but actually be malfunctioning in the present [15]. However, recall Franssen’s criticism of the normativity of evolutionary functions mentioned in the methods section. Many traits of an organism could be considered to be functional because of their evolutionary history, but would not qualify as functions in the everyday sense and are usually not evaluated with respect to norms. Franssen also sees a proliferation problem if, e.g., foxes are ascribed the function to hunt, eat and, thus, control the population of rabbits because they evolved in this way [18].

In any case, etiological accounts have problems with respect to the avoidance of epiphenomenalism and with novel functions: Functionality is exclusively determined by evolutionary development, and such historical facts are causally inert. Moreover, due to the necessity of having a selection history, there cannot be any functions in the first generation of a biological type, although the actual structure of the organs would be “functional” in the everyday sense, which is very counterintuitive. In addition, a certain body part may acquire new uses and functions during the evolutionary history of a species while the early history and, hence, the reasons for its existence remain the same [27]. Sea turtles, for example, use their flippers to bury their eggs in the sand; this seems to be one of the functions of their flippers. During the evolutionary history of the turtles, however, the flippers developed as a means for locomotion and only later acquired the digging function [32]. Peter Godfrey-Smith has argued that functional explanation has to focus on the recent history of species rather than on its distant evolutionary origins [33]. One of his arguments is that otherwise there would be no difference between functional explanations and evolutionary history, which are two distinct tasks according to Tinbergen’s famous four questions that biology has to answer [34]. Hence, if functions tell us anything about the “survival value” of a certain trait, the recent history of a species seems to be more important than its distant evolutionary past.

Furthermore, adaptionism is no longer seen as the only possibility how biological differentiations and functions can arise [35]. More generally, according to etiological accounts, the actual performance of a body part should not be relevant because the evolutionary history is the only thing that matters. Thus, functions seem to be mere epiphenomena of some causal history whereas, intuitively and methodologically, the essence of a function lies in what a thing can and is supposed to do now, which can be understood and discovered independently of how the thing came to be there.

### Intentional accounts of functions

As a third group of theories of functions we consider intentional accounts. They are tailored to accounting for the design functions of artefacts: A screwdriver can be said to have the design function to drive screws because it is produced with the plan to be used for this purpose. A design function, then, is not a property that inheres in the functional artefact, but it is the content of an ascription by an agent or a group of agents involving a plan about the future use of this artefact (or of artefacts of this type). They are made for a certain purpose, their function. Let us call this the planning account of design functions. On this account, the truth-maker of a function ascription is a plan. Houkes et al. have given an action-theoretic analysis of use and design claiming that the designer’s intentional plans are, in a sense, prior to both the design of an artefact and its use by a prospective user [36]. According to this approach, both use and design functions of artefacts are dependent on the designer’s (or the client’s) ends and on his plan for achieving these ends. On such an account, artefacts are to be described “as objects playing a role in the contexts of both use and design, contexts that are mediated by the communication of a user plan” [36]. (The more elaborate “ICE theory” of technical artefacts by Houkes and Vermaas [16] will be discussed in the next section as a further development).

Intentional accounts fare well with regard to the teleology criterion: Artefacts are produced in order to fulfil the function ascribed to them by their designer. They can distinguish well between essential and accidental features and they can account for normativity and innovation because all of these can be based on the intentions and expectations of the designer.

On the downside, design functions are grounded in designers’ function ascriptions and not in the physical structure of artefacts. Thus, an artefact could have any function independently of its physical structure and dispositions. (It will, though, not be able to realize its function unless it possesses a corresponding disposition to do so). One option to ameliorate this is to demand rationality of the designer: A rational designer would have justified (although, not necessarily true) beliefs about the components and their working together when he ascribes a function to the system, and would not assign functions not supported by the structure and dispositions of the components. However, it is not easy to see how this approach can be transferred to biology, for it seems to presuppose a rational and intentional Creator and looks almost like intelligent design theory, outrightly rejected by many (though not all) biologists, philosophers and theologians [37]. (We will later discuss a fictionalist approach that tries to overcome this problem.)

The intentional account can easily be modified in order to deal with the so-called use functions of artefacts (which would be classified as roles within the BFO framework). Use functions are directed at those activities that users actually use things for. (Cf., e.g., [28] for more on the distinction between design function and use function and [17] for problems of this distinction.) If I use my screw driver to open my paint cans, it has the use function to open paint cans. It has not been produced for this purpose; hence, the use function can differ from its design function, though it might be just the same. Moreover, one and the same thing can have many different use functions at different occasions. This account can also be extended to biomedical entities: If someone uses digitalis to kill his wife, he has a certain action plan that involves the participation of both a probe of digitalis and his wife with a certain intended outcome.

### The ICE theory

Houkes and Vermaas have developed a function theory for artefacts they call “ICE theory”, reflecting the fact that they include Intentional, Causal and Evolutionary elements in their account [16]. The main intentional element (I) is a use plan for the artefact that reflects the designers’ and prospective users’ intentions. The causal aspect (C) shows in their identification of function ascriptions with ascriptions of “physico-chemical capacities” or, in our terminology, dispositions that ensure the support of the function ([15]; p. 100):

A designer justifiably *ascribes* the disposition to V *as a function* to an artefact x relative to a use plan p for x, and relative to an account A, if and only if (I) the designer believes both that (I1) x has the capacity to V and that (I2) p leads to its goals due to, in part, x’s capacity to V; and (C) the designer can justify these beliefs on the basis of A.

The evolutionary aspect of the ICE theory is very weak and only relevant for function ascriptions by “passive”, non-designing users (as opposed to designers) and their use of artefacts because they need to have warranted information (by testimony) about the designer’s beliefs (I1) and (I2) and this is transmitted “historically”. The authors claim that this account does well according to their criteria, i.e. to (2) Restriction, (3b) Malfunctioning, (4b) Support and (5) Innovation. Due to the specifications of the originally intended use plan, accidental functions can be excluded and novel functions are no problem; and on account of the identification of functions with dispositions, there is no threat of epiphenomenalism: Since designers and users need to have the justified belief that the thing in question actually has this disposition in order to ascribe the corresponding function, the support criterion is satisfied and cases of wishful thinking are excluded. Note that this is an epistemic understanding of the “support” that the physical structure lends to functions. Although for many standard cases of function ascriptions the support criterion can be satisfied in this way, such rationality constraints do not completely exclude mad scientists and strange function ascriptions. They cannot guarantee that function ascriptions are always veridical, and it could happen that function ascriptions are rationally justified with respect to the available information but false.

Malfunctioning is still considered a difficult case for the ICE-theory by its authors. In many cases of malfunctioning, users ascribing an ICE-function may be wrong but justified because they are not aware that the artefact in question has lost the respective disposition. Users can also be fully aware of the lack of capacity because they know the artefact to be broken, but they assume that repair is possible and planned. Intuitively, we would ascribe the function to the broken artefact, but, according to the ICE definition, this would not be justified. Houkes & Vermaas distinguish having a disposition from its performance and use this for other cases of non-performance because necessary conditions for the realizations are not met, as in the case of a car with an empty fuel tank. Overall, they find it necessary to refer to a background of maintenance and repair to distinguish malfunctioning from non-functioning. Some damage of artefacts responsible for their malfunction can be repaired, so these are cases of (temporary) malfunction. However, in some instances, repair is not technically feasible; in such cases an artefact would definitely have lost its function.

### The fictionalist account

Another modification of the intentionalist approach is the fictionalist approach [38-40]. In pre-Darwinian biology, organisms and their parts were described as if they, too, were something created – either, allegorically spoken, by a personified Nature or by God as a creator. In the latter case, ascribing functions to biological entities could be conceived of as reading the mind of God before the act of creation and as a reconstruction of the reasoning underlying His creation; we mentioned this option in the context of the pure intentional account. This is no longer a viable account for modern science and much of the discussion about biological functions can be understood as finding some substitution for this intentional model. In the former allegorical case, we have something like an as-if parlance, which can be found, e.g. in Aristotle: Although Aristotle rejects the idea that the universe or life had a beginning in time, he often says that Nature has well organised her creatures [41]. We suggest to read this as a fictionalist or ‘as if’ way to talk about biological function, meaning: Were this plant or animal brought about by Mother Nature (a very intelligent designer), she would have done so for good reasons. Hence, the planning account can be upheld for biological functions with a small modification: The truth-maker of the ascription of a biological function is no actual plan, but a plan within the fiction of Mother Nature designing her creatures. The fictionalist account simply invites taking the intentional approach seriously as a model, i.e. a certain kind of fiction [42,43], without an ontological commitment to the existence of Mother Nature as a real person with beliefs and desires. That is, Mother Nature is the functional equivalent to extensionless mass-points in mechanics or rational utility maximizers in economics: They do not need to exist to make the theory in question an explanatory or predictive success. This way, the fictionalist account allows extending the intentional account to biological functions, while retaining the plain intentional account for artefacts.

The fictionalist account does well with respect to our list of criteria. Like in the original intentional account, functions feature in a teleological explanation (though some transfer is needed from the fictive design model to evolutionary reality). The fictionalist account can distinguish between accidental and essential functions; and it allows for normativity and malfunctioning. Novel functions can be accounted for if we situate the fictive designer at a later stage in evolutionary history faced with different evolutionary challenges. We can also give slightly more optimistic answers regarding the threat of epiphenomenalism and the support for the actual performance of a certain body part, as its physical structure is the most important evidence for the function of a body part and any informed judgement about the function of a part must account for how its structure supports this function. Finally, there is now a viable bridge from biological functions to artefactual functions, as intentional accounts fare well with the latter anyway.

## Discussion

### Formal ontological and philosophical accounts of functions

As should be clear now, the philosophical theories fare differently with respect to the criteria set out above (Table 2), and they do not always distinguish sharply between functions, roles and dispositions. However, BFO draws on the different function theories for its distinctions between the types of realizables. To begin with, BFO functions and BFO dispositions are determined by their causally relevant internal structure; this fits to the causal-role account, as it is said that the realization of a (biological) function “helps to realize the characteristic physiology and life pattern for an organism of the relevant type” [6]. The difference between dispositions and functions is founded on a historical (evolutionary) or intentional (design) component, respectively. Similarly, these intentional and historical criteria are used in BFO 2 as the specific difference of functions as opposed to non-functional dispositions. Thus, BFO functions are essential features of their bearers because of either an evolutionary or an intentional component, whereas functions according to a mere causal-role account would probably be classified as dispositions within BFO. What is called optional, accidental or “use functions” in the general debate are roles in BFO because they are not essential to their bearers. In fact, it is essential to BFO-roles that their bearers are not essentially playing these roles; this is particularly clear in the case of social roles.

**Table 2:** Evaluation of philosophical function theories

	**Causal role accounts**	**Etiological accounts**	**Intentional accounts**	**ICE account**	**Fictionalist account**
**(1) Teleology**	-	+	+	+	+
**(2) Accidental/essential**	-	+	+	+	+
**(3) Normativity/Malfunctioning**	-	+	+	+	+
**(4) Support/No epiphenomalism**	+	-	-	+	+
**(5) Novel functions**	+	-	+	+	+
**(6) Continuants**	+	+	+	+	+
**(7) Bridging of domains**	+	+	-	-	+

### Function and homology

When explicating the distinction between functions, roles and definitions in BFO, Arp and Smith define biological functions as follows:

“A biological function is a function which inheres in an independent continuant that is (i) part of an organism and (ii) exists and has the physical structure it has as a result of the coordinated expression of that organism’s structural genes.” [6]

Philip Lord [44] points out that several *prima facie* bio-functions are not captured by this definition because of its restriction to organism parts on the one hand and to gene expression as grounds for the structure on the other hand. His counterexamples are molecular functions and functions of whole organisms. Lord gives an alternative definition:

“A biological function is a realizable entity that inheres in a continuant which is realized in an activity, and where the homologous structure(s) of individuals of closely related and the same species bear this same biological function.” [44]

Lord claims that his definition is recursive rather than circular, despite the occurrence of the word “function” in the definiens. While this can be considered to be a problem on its own, his suggestion is also open to counterexamples when it comes to more recently acquired functions like the sea turtle’s flippers, which are used to bury their eggs in the sand (see above), but have homologues in other species that have (only) the function of locomotion. Lord realizes this when trying to distinguish functions from roles: A human can walk on his hands, but the function of the human hand is not walking as most humans do not walk on their hands, whereas the hand’s homologous structures in other primates do have this function. Therefore in humans, the hand may have the role, but does not have the function walking. Hence he concludes that among the instances of realizables that are realizables for the same type of process can be both roles and functions depending on the species the realizable’s bearer belongs to. This presents a problem for the distinction between functions and roles. We will come back to this distinction in due course, but it should be noted that this problem exists for Lord mainly because of the reference to homologues in other species in his function definition. To put it bluntly: Had evolution stopped after the first species, according to Lord’s definition, there would not have been any biological function at all. If we look only at humans, we would not suppose that the function of the human hand is to walk with, but that it can assume such a role under certain circumstances for appropriately trained individuals. Therefore, it seems that connecting functions by definition with homologues in other species (and *their* respective functions) does introduce more difficulties than it solves, even if homologues are of huge importance heuristically.

### Function vs. role

Let us in more detail review the differences between functions and roles. Lord [44] states that in actual biomedical ontologies, this distinction between functions and roles is hardly observed. Lord cites OBI, the Ontology for Biomedical Investigation [45], as an example that ignores this distinction. The context dependence does not seem sufficient to distinguish functions from roles. Dumontier has claimed that on the level of proteins and molecules the distinction between functions and roles becomes redundant: “The difference between functions and roles is not particularly obvious in molecular systems and may in fact be redundant. For instance, the function of an enzyme is to catalyze a reaction […] Every time a protein executes such functionality, it necessarily realizes the enzyme role” [46]. Consequently, Dumontier uses only roles in his analysis of molecular reaction. This seems appropriate because the very same type of protein can have the enzyme role in one reaction type and a different (e.g. substrate) role in a different reaction type. In our approach, we would ascribe several dispositions to the protein as prerequisites to the different realizations that would correspond to the roles of enzyme etc., so the protein would have dispositions and roles, but no functions. Also, subrelations of the “hasParticipant” relation could be employed when the different participants of a reaction are classified according to their roles like “hasSubstrate”, “hasProduct” or “hasCatalyst”. Note that the inverse relation “participatesIn” and its subrelations cannot be used for this purpose because the same type of molecule can have different roles in reactions and, thus, it will generally not be true that all molecules participate in some reaction type.

Accordingly, many so-called functions in biomedical ontologies are, strictly speaking, roles. Also the so-called use functions of artefacts are roles. To come back to our function criteria, roles clearly fail the first two, Teleology (1) and Restriction (2). They fail the Teleology criterion because a role-bearer is not created for the playing of a mere role. This seems obvious in the case of artefact use cases not intended by the designer, i.e. using a chair to stand on to change a lightbulb, or social roles. Similarly, rabbits have not been selected for being food for foxes, though they may serve the role of fox food. Roles also fail the essential-accidental distinction; as can be seen by the examples given so far, roles are all accidental in a sense. The other criteria are less clear. Malfunctioning can, in some cases, be applied to roles, especially to social roles. In other cases, there is no plausible norm for the role to be evaluated against, as in cases of accidental use or misuse, because an artefact that is explicitly used for an unintended purpose will not be expected to perform optimally. Roles also usually must have some physical support in the dispositions and abilities of their bearers to have successful realizations. For example, a screwdriver can only serve the role as a makeshift chisel, because of its shape, its hardness etc.

### Functions vs. dispositions

Now, what does our review tell us about the relation between functions and dispositions? A common philosophical position thinks of dispositions as a certain type of properties [7,8,47]. According to this position, a disposition is a causal property that is linked to a realization, i.e. to a specific behaviour or process which the individual that bears the disposition will exhibit under certain circumstances or as a response to a certain trigger. Something is water-soluble if it can dissolve in water. In this fashion, dispositions establish a link between independent continuants (stable things) and occurrents (processes). The fundamental connection is the following: Continuant type S has disposition type D for a realization type P and, in case some token p of P occurs as the realization of an instance of D, then an instance s of type S is both the bearer of the disposition d and a participant of this process instance p. The category of dispositions is often treated as a special kind of dependent continuants that are linked to a process of realization by a respective formal relationship [6-8].

Some, but not all of the positions surveyed above are explicit about the relations between functions and dispositions, and those that are do not agree on this matter. While Cummins writes that “if something functions as a pump in a system […] then it must be capable of pumping” and that “to attribute a function to something is, in part, to attribute a disposition to it” ([26]; p. 757–8), Millikan states that a thing’s having a function “has to do not with its powers but with its history” ([31]; p. 17). On a first superficial view, the idea that functions are, in fact, dispositions seems to be at least compatible with most of the positions reviewed: According to the causal role accounts, functions can be identified with dispositions quite easily. Millikan’s resistance notwithstanding, other authors and BFO 2 combine an etiological account with the claim that functions are evolutionarily acquired dispositions [9,33]. Intentional accounts can argue that functions are specially designed dispositions and the authors of the ICE account actually state that function ascriptions are special kinds of disposition ascriptions. More scrutiny, however, will show that the criteria (3a) and (3b), i.e. normativity and malfunctioning, require us not to subsume functions under dispositions.

### Are functions dispositions?

We have seen that among others, both BFO 2 and Houkes and Vermaas conceive of functions as special dispositions (or of function ascriptions as special ascriptions of dispositions). We will now apply some of the criteria for functions above to try to capture also the prima facie differences between functions, roles and dispositions. Dispositions can be blocked or incompletely realized, but their bearers are not evaluated in a normative fashion. We usually do not evaluate the realization of a disposition like inflammability; although, we certainly have rational expectations about the “performance” of the bearer of a disposition and rely on them in our intentions. Suppose that someone wants to start a campfire to get warmth and all available wood is wet. In such a case, the wood will only burn with difficulty and one will in fact evaluate the available wood negatively with respect to its inflammability, but only because one ascribed a use function (i.e. a certain role) to the wood before. The having of a disposition need not explain the existence of the disposition-bearer, so they generally fail with respect to the teleology criterion, although there might be some cases where the having of a disposition does explain the existence of its bearer. There are materials (like alloys or textiles) that are specifically created for their dispositions because the artefacts made from the material will need those dispositions to fulfil the functions they are intended for. Thus, in these cases, some chunk of the alloy comes into existence because it was designed to have a certain disposition. Could this be taken as an argument for functions as special dispositions? We do not think so. The teleological dimension, like the normative one, applies to dispositions only in the case of artefacts or in connection with intentional use; that is, in contexts that have a teleological component (usually intentional) anyway.

Peter Kroes has also argued against seeing functions as a type of dispositions because dispositions lack the normative dimension we need for functions [48]. Although his argument relies on Carnap’s dated analysis of dispositions, it does show the salient difference and could probably be adapted within a more sophisticated analysis of dispositions.

Dispositions are usually ascribed because of their realization (their performance). They can be distinguished from functions according to the criterion (4a) because there is no tension between current performance and normativity for dispositions. Other than functions, dispositions do not face the threat of being mere epiphenomena.

One further central difference between dispositions on the one hand and functions and roles on the other hand seems to lie in their context-dependence. Continuants may lose or acquire dispositions, but not without fundamental changes within the bearer. In contrast, many functions can be performed by different types of bearers and an object may have different functions in different contexts without any change in itself. Chopsticks, for example, have the function to support eating. Similar sticks found in the woods do not have any such function, though they may have the very same physical structure and, hence, the same dispositions. Dispositions, that is, are purely internally grounded, while the function of the chopsticks is a historical property due to the way this artefact has been produced. At the other end of the spectrum, social functions and roles are externally grounded; that is, they are dependent on the respective context, relational and historical properties and mostly independent from the physical structure of their bearers. Biological functions like those of organs, enzymes etc. lie somewhat in between these extremes, as an entity can usually perform several functions in a certain range of contexts. They are objective systemic functions in the sense mentioned above and not merely ascribed by an agent; their context-dependence is fixed by the functional hierarchy of the respective physiological system. An organ like the liver has many functions, like the production of bile, glycogen storage, cholesterol synthesis etc., but all these are fixed by the respective physiological systems in which the liver and its products are functionally involved. They are not as arbitrary or flexible as the screwdriver that can serve the use functions (i.e. roles) of a can opener or a weapon.

### Do functions depend on dispositions?

For all of these reasons, we should assume that functions are not identical to dispositions. Nevertheless, even if they are distinct entities, functions could ontologically depend on dispositions. The support criterion suggests indeed that, in some sense, functions could be “based” on dispositions. On the intentional account, functions of artefacts are clearly independent from the dispositions of their bearers; due to the fallibility of human designers, the one could easily occur without the other. From the point of the fictionalist extension of the planning account to biological functions, however, the existence of a function implies the existence of the corresponding disposition in typical cases if we transfer the usual assumptions of God’s omniscience and benevolence to our fictional designer. Nonetheless, even if a biological function is typically accompanied by a disposition, this concurrence is not universal, as proven by malfunctioning.

In our view, the dispositions are part of the internal structure of a thing; they determine whether it can fulfil the respective function in a given context. Johansson [49] calls this the “substratum” of a function. While the function itself is independent from its substratum, its realization depends on its existence. This dependence can be a generic one because sometimes different dispositions or structures can ground the same function: E.g. the cooling function of a cooler can be implemented in different technical setups [49].

As we know biological functions only through their actual realizations, we would have no reason to ascribe them unless instances of a certain kind typically displayed that behaviour and, a fortiori, possessed a corresponding substratum disposition. How would we know the biological function of, say, a heart, if hearts did not typically have the disposition to pump blood and did not typically realize this function? Thus, there should be some evidential connection between the function and the disposition of the organ. This way, we can meet the Support requirement (4) by giving it an epistemic interpretation: The discovery and ascription of a function is epistemically supported by the preliminary discovery and ascription of dispositions in the same or other instances of a certain species.

On the other hand, many diseases like, e.g. heart insufficiency, are characterized by the very contrast between functions and the lack of corresponding dispositions and so is malfunctioning in general. Malfunctioning artefacts or diseased organs are characterised by the loss of the disposition to fulfil their function. E.g. a lung with a carcinoma will still have the function to serve as an oxygen provider for the body, but the function may no longer be realized because the corresponding disposition (to be able to serve as an oxygen provider for the body) is no longer present. Such an account of malfunctioning works because (and only if) the function is ontologically independent of the disposition. From this point of view, the task of medicine is to restore the disposition matching to the function, such that the organ would be (fully) functional again. In such a fashion one can also account for healing processes: A healing process, then, consists of restoring a disposition where there is a function without its corresponding disposition. We conclude that the corresponding disposition is only necessary for the *realization* of a function, not for the function itself. Since in biological (and many artefactual) cases we can evaluate the performance of token functions with respect to what is a normal realization for the function type and because the normal realization is dependent on the corresponding disposition, we have a correspondence of function and disposition at the type level or for prototypical tokens. However, this is to be distinguished from a token-level dependence of the function on the corresponding disposition. If we want to accommodate malfunctioning, we should reject the latter.

As a source of the normativity of functions, we can identify the type membership of an instance of a function bearer. Being a token of a type involves an evaluative dimension [32,50,51]. Therefore, in function ascription, the attribution of a function to a type of entity takes preference over the ascription to a token. A token is supposed to have a function and be able to perform it successfully because it belongs to a certain type.

A further reason not to treat functions as special dispositions is the following difficulty: In formalised fashion, type-level relations like “hasFunction” are usually defined by universal quantification over their instances using the corresponding token-level-relation [52]. As said above in the “normal” or paradigmatic case, a function of an entity comes along with the disposition to perform this very function, but as we want to allow for the possibility of malfunctioning tokens that have lost the corresponding disposition and, consequently, perform the function insufficiently or not at all, we cannot assert a token-level dependence of disposition and function for all instances of a type. One option here would be to distinguish between canonical and non-canonical entities within a type of function-bearers. Such a distinction is used, e.g. with respect to anatomical structures in the BioTop ontology [53]. If one follows this approach, all instances of the canonical type have the corresponding disposition and do (or would) function adequately, so for this subtype the standard definitional procedure works. The instances of the non-canonical subtype will not be ascribed the disposition, but only the function.

### Recommendations

We can summarize the discussion by suggesting a new classification schema for the three realizables function, disposition and role. It concurs with BFO 1.1.1 in treating functions as siblings of dispositions rather than special dispositions as in BFO 2. It makes use of two independent criteria:

• Structure [6]: Does the realizable supervene on the internal structure or is it externally grounded?

• Rigidity [54]: Is the realizable essential or accidental to its bearer?

Structure and Rigidity correspond to two flavours of (non-)optionality. While Structure deals with (non-)optionality given the physical structure of the bearer, Rigidity deals with (non-)optionality given the “essence” of the bearer (i.e. given the kind of thing it is). Thus, we end up with a cross classification of realizables presented in Table 3.

**Table 3:** A new cross-classification of realizables

	**Internally grounded (= non-optional given the physical structure)**	**Externally grounded (= optional given the physical structure)**
**Essential** (= non-optional given the bearer)	Essential disposition	Function
**Accidental** (= optional given the bearer)	Accidental disposition	Role

We will start the elucidation with Structure. A realizable can be optional given a certain physical structure of its bearer. All realizables that are externally grounded, i.e. grounded in some context, are optional in this sense, e.g. all roles. In contrast, dispositions are internally grounded, based on the bearer’s physical structure and, therefore, not optional given the bearer’s physical structure. A few clarifications seem in order: Internal grounding does not require that there is a one-to-one-correspondence between a disposition type and a structural type on which it is based. Various instances of one and the same type of dispositions can be based on quite different physical structures. Fragility, for example, may be based on the molecular structure of dry wood as well as on the structure of glass, respectively. Like many other dispositions, fragility can be structurally constituted in multiple ways, but in all of these ways the disposition’s base is a structure literally internal to the bearer of the disposition, and it is independent of the context or the realization conditions.

There is a debate whether dispositions need laws of nature to get “connected” to their realizations or whether laws of nature are actually based on dispositions, so the latter are ontologically more basic than laws [55]. We will not enter this debate here, but we note that even if dispositions depend on laws of nature (whatever laws of nature are), this will not be an ontological dependence that would force us to reject the claim that dispositions are internal to their bearers.

Second, a realizable can be optional given the essence of its bearer. Roles are optional in this sense, and since a bearer can gain and lose dispositions, some dispositions are also optional in this way. Note that in this case they are not optional given the physical structure, but optional given the bearer: The same bearer can survive the acquisition of such a disposition (by learning, training, modification, etc.) and it may lose it without ceasing to be (by forgetting, wear out, modification, etc.), although the bearer has to change its physical structure in order to gain or lose dispositions. However, not all dispositions are optional for a given bearer. Some dispositions, like the disposition of a proton to attract electrons, are essential: Losing this disposition would imply that the proton ceases to be a proton, i.e. that it ceases to exist. There might be only a few essential dispositions and they might be restricted to the domain of fundamental physics. For this reason, we acknowledge their existence, but do not encourage the introduction of separate ontological categories for accidental and essential dispositions, respectively. Functions, too, are essential for their bearers: Given the essence of being a heart, it is not optional to have the function to pump blood; and given the essence of being a screwdriver, it is not optional to have the function to manipulate screws. This is why screwdrivers are made; it is their origin, without which they would not be the things they are. Without these functions they would not be hearts or screwdrivers, but rather something else. This notion of essentiality for functions seems to be shared by Kitamura and Mizoguchi with respect to artefacts [22]. In a similar way, both the etiological and the system account can argue that the bearers of biological functions come into existence in order to fulfil these functions; the fictionalist account will assert similar things relative to the respective design fiction.

Functions are externally grounded. We argued that there are good arguments not to treat functions as dispositions, nor to make functions dependent on dispositions. This distinction is our central disagreement with the BFO 2 suggestion discussed above. We also define roles in a rather narrow way (following BFO, but diverging from [13,28,56] and others): On our account, roles are never essential for its bearer. Hence, we think that assigning an essential “breather” or “eater” role to a human being is a loose way of speaking and not to be taken ontologically serious. Breathing and eating are processes, not functions or roles. True, humans have to participate in breathing and eating processes on a regular basis, but there is no need to add to it by postulating a breather role or an eater role for human beings. One could treat participation (or rather the property of being a possible and viable participant) in processes as a role in a very general sense. Starting from the original BFO suggestion, however, the category name “Role” is used in a more specific way that is distinct from participation. In our view, participation is an ontological relation [57] (which would be modelled as an object property in OWL), whereas a role is a realizable; that is, a (specifically) dependent continuant. The realization of a role, of course, involves the role-bearer standing in the participation relation of a realization process of the type that is implied by that role. One of the main points of introducing roles is to have the option to model these less strict relationships with a modal character that cannot be modelled by stating the participant relation which only concerns the actual participation. Therefore, the so-called “use functions” (cf. above) are to be classified as roles in our classification scheme, in agreement with the BFO conception of roles.

## Conclusions

We surveyed several accounts for the analysis of functions, both for biological functions and for engineered functions, and we evaluated them with respect to a list of criteria compiled from the literature. While some of these theories treat functions as a subclass of dispositions, this cannot account for the normativity connected with function ascriptions and for the possibility of malfunctioning. We suggest a new classification of realizables by means of two independent criteria yielding four subcategories: essential dispositions, accidental dispositions, functions and roles. Treating functions as a category in its own right solves the problems of normativity and malfunctioning.

On this account, functions are not only disjoint from dispositions, they are also ontologically independent from dispositions. Functions are, however, normally and mostly accompanied by corresponding dispositions. Moreover, there cannot be a realization of a function without there being a realization of a matching disposition. It is for these reasons, why it is so difficult to distinguish between these categories. Malfunctioning, however, requires them to be distinct categories: It happens in case a function is present but the corresponding disposition is lacking.

## Competing interests

The authors declare that they have no competing interests.

## Authors’ contributions

Both authors contributed equally to the present work. Both authors read and approved the final manuscript.
